# The Chinese Herb* Jianpijiedu* Contributes to the Regulation of OATP1B2 and ABCC2 in a Rat Model of Orthotopic Transplantation Liver Cancer Pretreated with Food Restriction and Diarrhea

**DOI:** 10.1155/2015/752850

**Published:** 2015-11-17

**Authors:** Baoguo Sun, Yan Chen, Ting Xiang, Lei Zhang, Zexiong Chen, Shijun Zhang, Houming Zhou, Shuqing Chen

**Affiliations:** Department of Traditional Chinese Medicine, The First Affiliated Hospital, Sun Yat-sen University, 58 Zhongshan Road II, Guangzhou 510080, China

## Abstract

Traditional Chinese Medicine* Jianpijiedu* decoction (JPJD) could improve the general status of liver cancer patients in clinics, especially the symptoms of decreased food intake and diarrhea. In this study, our results showed that the survival rate of the liver cancer with food restriction and diarrhea (FRD-LC) rats was lower than the liver cancer (LC) rats, and the tumor volume of the FRD-LC rats was higher than the LC rats. It was also shown that the high dose of JPJD significantly improved the survival rate, weight, and organ weight when compared with FRD-LC-induced rats. Moreover, JPJD administration upregulated the mRNA and protein levels of ABCC2 and downregulated the mRNA and protein levels of OATP1B2 in liver tissues. However, opposite results were observed in the cancer tissues. In conclusion, the study indicated that the Chinese Medicine JPJD could contribute to the rats with liver cancer which were pretreated with food restriction and diarrhea by regulating the expression of ABCC2 and OATP1B2 in liver tissues and cancer tissues.

## 1. Introduction

Liver cancer is characterized by high mortality and poor prognosis. The liver microenvironment, including signaling by noncellular factors, is now recognized as an important aspect of liver cancer pathogenesis, progression, and treatment response [1].

Decreased food intake and diarrhea are the most common clinical symptoms in patients with liver cancer. Intractable diarrhea appears as the first symptom in many cases [2, 3], while decreased food intake and diarrhea induced by antiliver cancer drugs such as sorafenib are also frequently observed [4]. Long-term decreased food intake or diarrhea will inevitably result in malnutrition [5], which results in poor prognosis in chronic liver diseases such as liver cancer [6, 7]. Reduced food intake may impair immunity [8, 9] and preoperative fasting will increase the risk of blood cancer cell metastasis [10]. Therefore, decreased food intake and diarrhea are important clinical symptoms of liver cancer that may greatly alter disease progress and prognosis. However, little is known about the influences of decreased food intake and diarrhea on the effect and mechanism of liver cancer development.

Transmembrane transporter proteins, which are widely distributed on cellular surfaces, are important for the absorption, distribution, and excretion of drugs and other xenobiotics that cannot be freely transported through cellular membranes. Organic anion transporting polypeptides (OATPs) are members of the solute carrier transporter superfamily, which are responsible for transporting extracellular substances into the cell [11]. OATP1B1 and OATP1B3 which are distributed in the liver are mainly responsible for transporting the extracellular substances into the liver cells from the blood and regulating metabolism and gene expression [12]. Some studies illustrated that OATP1B3 also showed high expression in some tumor tissues and cells, and there was a close relationship between OATP1B3 and cancer [13, 14].

In contrast to OATPs, ATP-binding cassette transporters are responsible for transporting intercellular substances out of the cell [15]. OATP1B2 and ABCC2 are located in the liver and they are crucial regulators of the liver microenvironment. Dietrich et al. found that the high expression of ABCC2 could reduce the carcinogenic effects in rats [16]. Li et al. found that the high expression level of ABCC2 could stop chemotherapy drugs to act on the liver cancer cells, because ABCC2 could transport the toxic substances (such as cytotoxic drugs) out of the cells [17]. It indicated that the body's susceptibility to disease could be decreased through enhancing the expression level of ABCC2 so that the toxic substances could be transported out of the cell from the human body.

However, little is known about the expression of OATP1B2 and ABCC2 in the liver and during liver cancer. Furthermore, previous studies have provided no direct evidence of the roles of OATP1B2 and ABCC2 during the pathological progression of liver cancer. Our recent studies [18] revealed that OATP2A1 played a pivotal role in the development of liver cancer. The general status of liver cancer patients was improved by [19]* Jianpijiedu* decoction (JPJD). However, further research is needed to determine whether JPJD positively impacts the liver microenvironment during liver cancer by regulating the levels of OATP1B2 or ABCC2. And our previous studies [18, 20] have shown that the expression levels of OATPs such as OATP2A1 and OATP2B1 in the liver could be altered in rats suffering from food restriction and diarrhea.

Therefore, this study was designed not only to investigate the expression of OATP1B2 and ABCC2 in the liver and hepatoma tissues of an orthotopic transplantation liver cancer rat model which was pretreated with food restriction and diarrhea (FRD-LC), but also to investigate the influential role of JPJD in the expression of OATP1B2 and ABCC2.

## 2. Materials and Methods

### 2.1. Ethics Statement

This study was approved by the First Affiliated Hospital of Sun Yat-sen University Animal Experiments Ethics Committee (protocol number: [2013] 149), and the protocols were performed in accordance with the Guide for the Care and Use of Laboratory Animals published by the US National Institutes of Health (NIH Publication number 85–23, revised 1996). Animals were purchased from the Experimental Animal Center of Sun Yat-sen University. Animal care and experiments were conducted under institutional guidelines and food and water were given ad libitum. All surgeries were performed under sodium pentobarbital (90 mg/kg, i.p.) anesthesia to minimize the pain caused by the experimental procedure. And the electric heating pad was used to warm the animals while they received the anaesthetics or to accelerate the recovery from anaesthetics. Animals were sacrificed by cervical dislocation under anesthesia in line with the euthanasia principle. We recorded the body weight of each rat on an empty stomach and the appearance of the health status at 0800 h every day. When the rats reached the ethical limits of animal care (apathy, curl-up, body weight loss 20%, chills, or ascites) during the experiment procedure, they were sacrificed by cervical dislocation under anesthesia immediately in order to relieve suffering.

### 2.2. Experimental Animals and Cells

The Wistar rats and BALB/c nude mice were provided by the Experimental Animal Center of Sun Yat-sen University (protocol number: SCXK (Beijing) 2012-0001 and SCXK (Guangdong) 2008-0002, resp.). The 105 male Wistar rats aged 3 weeks and 4 male BALB/c nude mice aged 2 weeks used in the study were housed in the Specific Pathogen Free (SPF) lab (environmental facilities license SYXK (Guangdong), 2010-0108). The Walker-256 cells were provided by Experimental Animal Center of Sun Yat-sen University, which obtained the cell line from the Type Culture Collection of Chinese Academy of Sciences (Shanghai, China). The Walker-256 cell line was intraperitoneally (IP) administered to the rats. After 7 days, the rats were submitted to euthanasia, and the ascitic fluid was collected and centrifuged for 10 min at 1000 rpm at 4°C. Before euthanization, the rats appeared emaciated and presented vast ascitic signs. The supernatant was discarded, and the precipitate was resuspended in 1 mL suspension medium of PBS (16.5 mmol phosphate, 137 mmol NaCl, and 2.7 mmol KCl). The cells were counted with Hemocytometer, the viability of the cells was assessed using the Trypan blue exclusion method in a Neubauer chamber, and approximately 1 × 10^7^/mL Walker-256 cells suspension was prepared [21].

### 2.3. Medicines

All the Chinese herbal medicines used in this study were provided by the Guangzhou Chinese herbal medicine company. The mirabilite solution consisted of 2 g thenardite, which contained 99.0% Na_2_SO_4_, and was dissolved into 1 mL of saline and UV sterilized. The concentrated JPJD cream, which was composed of* Codonopsis*,* Poria*,* Atractylodes*,* Licorice*,* Bupleurum*,* Curcuma, and Scutellaria barbata* according to the established ratio [7, 8], was concentrated using water extraction, and the volatile oils were collected at the Guangzhou University of Chinese Medicine Science and Technology Industrial Park Co. The JPJD extract was diluted into an aqueous cream at a concentration of 2 g of crude drug per milliliter.

### 2.4. Reagents and Primers

The reagents for the fluorescence-based quantitative RT-PCR reactions and the Western-blot reactions and Immunohistochemistry detection were prepared using the manufacturer's instructions. The antibodies from goat for ABCC2 (SC-5770), the antibodies from rabbit for OATP1B2 (SC-134460), donkey anti-goat IgG-HRP, and goat anti-rabbit IgG-HRP were provided by Santa Cruz Biotechnology, Inc. The Rabbit anti-GAPDH (AB-P-R001) antibody was provide by Hangzhou Goodhere Biotechnology Co., Ltd. The following probes and primers were used for mRNA analysis: ABCC2 forward primer: 5′-CTG TCC ATG CTT CCC ATG GT-3′, reverse primer: 5′-GGC GAA TGG CAG ATG TGT CT-3′, probe: 5′-FAM-CTC ATC GAT CCT CCA GGC CAG TGT TT-BHQ1-3′; OATP1B2 forward primer: 5′-CAG TGG CAG GCT TAA CAA CCT-3′, reverse primer: 5′-GGA TCC CAT GTG TTC GTT GAG-3′, probe: 5′-FAM-TGG AGA ACA AGG TCC TTG CTG ACT-BHQ1-3′; and the housekeeping gene GAPDH forward primer: 5′-AGG GCT GCC TTC TCT TGT GA-3′, reverse primer: 5′-AAC TTG CCG TGG GTA GAG TCA-3′, probe: 5′-FAM-CCA TCA ACG ACC CCT TCA TTG ACC TC-BHQ1-3′. The probes were synthesized using an Invitrogen ABI 3900 high-throughput DNA synthesizer.

### 2.5. Experimental Design

#### 2.5.1. Establishment of the Rat Model of Intrahepatic Tumor Implantation-Induced Liver Cancer

2 × 10^6^ Walker-256 cells were transplanted subcutaneously at the neck of the 4 nude mice. After the mice were anesthetized with pentobarbital sodium (Sigma-Aldrich, 90 mg/kg, i.p.), the tumors were harvested after they were cultured to a diameter greater than 0.5 cm [22]. The tumor tissues were cut into pieces (approximately 1 mm^3^) after removing the necrotic tissues.

While the Wistar rats were anesthetized using pentobarbital sodium (90 mg/kg, i.p.), a small subxiphoid midline incision was made to expose the left lateral lobe of the liver. A small superficial incision of the liver was made, and a cube of hepatic tumor tissue was implanted. Soon after hemostasis by compression with a swab, the liver was not sutured and the abdominal incision was closed with 4-0 sutures layer by layer. The rats were kept warm on a heating pad until they regained consciousness [23].

#### 2.5.2. Establishment of the FRD Model

The rats were housed individually at 23 ± 1°C with a 12 h-12 h light-dark cycle and a feeding regimen of tap water ad libitum and alternate-day food restriction (the animals were permitted to take food from 9 a.m. to 9 a.m. on the following day) [24]. The rats received no treatments except for the gavage of mirabilite solution at a concentration of 0.25 g·mL^−1^ (1 mL·100 g^−1^ weight) daily for 29 days.

#### 2.5.3. Syndrome Criterion of the FRD Model [25]

The syndromes are as follows:

- being apathetic, lying activities, laziness, and clustering together,
- reduced eating and weight loss,
- loose feces.

The syndrome criterion of the FRD model was in order to select the animals which met the requirements of a successful FRD model. If all of the syndromes listed appeared in a rat, this rat would be included and receive the following interventions afterwards.

#### 2.5.4. Animal Grouping

Wistar rats (*n* = 105, 5 rats in 1 cage) were housed with free activity and access to water and food in the standard SPF animal lab at a constant temperature of 22 ± 1°C and a 12-hour light-dark cycle. One week after entering the lab, all rats were randomly divided into 7 groups (A: normal group, B: LC group, C: FRD group, D: FRD-LC group, and E, F, and G groups: administration of low (9.375 g·kg^−1^), medium (18.75 g·kg^−1^), and high (37.50 g·kg^−1^) doses of JPJD in FRD-LC treated rats, respectively, with *n* = 15 in each group). The rats in Group A were housed with free activity and access to water and food and received no treatment except for a saline gavage (1 mL·100 g^−1^ weight) once a day, while the rats in Groups C, D, E, F, and G received a drug gavage. The FRD model was established in Groups C, D, E, F, and G for 29 days. The samples in Group C were collected 1 day after the model was successfully established. Similarly, after 7 days of free feeding, the LC model was established as previously described in Groups B, D, E, F, and G. The rats in Groups E, F, and G were intragastrically administered JPJD at low (9.375 g·kg^−1^), medium (18.75 g·kg^−1^), and high (37.50 g·kg^−1^) doses, respectively, once a day for 42 days. The remaining rats received no treatment except for a saline gavage (1 mL·100 g^−1^) once a day for 42 days while the LC model was established. The animals' condition was monitored every day in case of unpredictable death. The survival times, weights, liver weights, and tumor volumes of the rats were recorded during the 42-day procedure.

#### 2.5.5. Sample Collection

Rats were sacrificed by cervical dislocation after they were anesthetized using pentobarbital sodium (90 mg/kg, i.p.), and a long midline incision was made to expose all the lobe of the liver, the spleen, and the thymus. The liver, the spleen, and the thymus were harvested and weighed. Then the tumor was extracted from the left lateral lobe of the liver and weighed. After those two samples were extracted from the right lateral lobe of the liver and tumor tissue, each sample was approximately 5 × 5 mm. One part of the samples which would be used for RT-PCR reaction and Western-blot detection was placed in freezing tubes and preserved within 15 minutes when they were placed in a −80°C refrigerator, and the other parts of the samples which would be used for immunohistochemical studies were fixed with 4% paraformaldehyde and embedded in paraffin. Meanwhile, when the rats reached the ethical limits of animal care (apathy, erected back hair, the rats being weighed every day at eight o'clock, and body weight loss of 20%, chills, or ascites), they were sacrificed by cervical dislocation under anesthesia immediately in order to relieve suffering and the samples were harvested. The rats would suffer from cachexia if the body weight lost was more than 20%. So once their body weight loss reached 20%, they were sacrificed immediately and the samples were harvested according to the ethical limits [26].

### 2.6. Fluorescence-Based Quantitative RT-PCR Reactions

Six rats from Group A and 6 intrahepatic tumor-implanted rats with tumor sizes greater than 1 cm^3^ were chosen randomly to analyze the levels of OATP1B2 and ABCC2 mRNA in the liver and cancer tissues. The total RNA was extracted using Trizol, and the DNA was obtained by reverse transcription with a BIO-RAD quantitative PCR instrument. The quantitative standard curve was prepared based on a gradient of positive standard with a negative control of sterile double-distilled water. The following PCR conditions were repeated for 40 cycles: 93°C for 2 min, 93°C for 15 s, 55°C for 25 s, and 72°C for 25 s. The reference gene was GAPDH. OATP1B2 and ABCC2 mRNA expression levels were calculated using the following equation: A (target gene relative copies) = B_1_ (target gene)/B_2_ (reference gene). By double ΔCt value method, target gene relative expression level = 2^−ΔΔCt^, ΔCt = Ct (target gene) − Ct (reference gene); ΔΔCt = ΔCt (treatment group)  −  ΔCt (contro1 group) [27].

### 2.7. Western-Blotting

Six rats were chosen randomly from each group to detect the OATP1B2 and ABCC2 proteins in the liver and cancer cells. The following steps were used for Western-blotting [28]: total protein extraction, SDS-PAGE gel preparation, protein sampling, electrophoretic separation, film transfer, blotting rig closing, first and secondary antibody incubation, and color rendering. The protein expression levels were analyzed using a semiquantitative method of relative optical density that was calculated using the integrated optical density of the target band divided by the integrated optical density of the reference band.

### 2.8. Immunohistochemistry

Paraffin-embedded tissues were sectioned into slices of 3 *μ*m in thickness. Slices were put into xylene for 10 min twice as soon as they were baked 2 h at 80°C. The sections were then treated with a solution of 3% H_2_O_2_ for 10 min at room temperature after being dehydrated through alcohol. Then the sections were treated with microwave in citrate buffer (0.01 mol/L, PH 7.8) for 10 min in order to repair antigen. Slides were then blocked with 10% normal goat sera in TBS for 15 min at 37°C followed by incubation with diluted primary antibody overnight at 4°C. Sections were then incubated with secondary antibody for 15 min at 37°C, followed by DAB substrate detection for 5 min at room temperature. After washing with distilled water the sections were counterstained with Harris hematoxylin solution dehydrated, cleared in xylene, and mounted with synthetic resin mounting medium and coverslip. Brown-yellow stain was positive staining [29].

### 2.9. Statistical Analysis

The data were analyzed using the Statistical Product and Service Solutions (SPSS16.0) software package for Windows. The survival time was analyzed using the Kaplan-Meier and Log-Rank (Mantel-Cox) test. The normally distributed data were analyzed using one-way ANOVA, and the abnormally distributed data were analyzed using independent sample rank sum tests. *P* < 0.05 was considered to indicate a statistically significant difference.

## 3. Results

### 3.1. Survival Analysis

**Figure 1:**
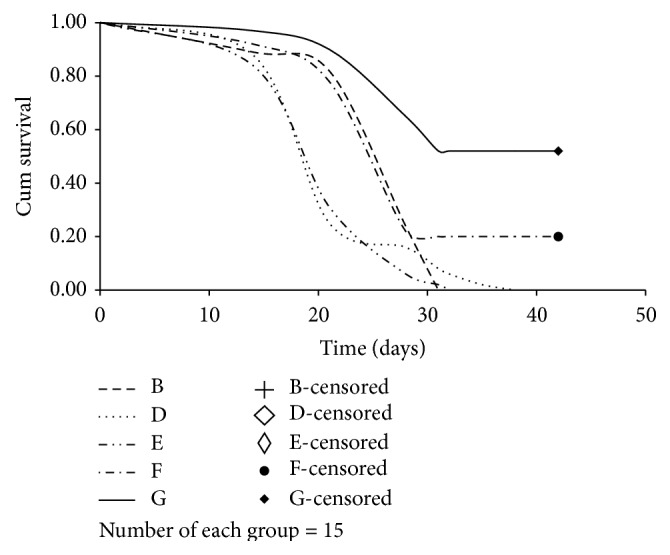
Survival analysis. Kaplan-Meier and Log-Rank (Mantel-Cox) Pairwise Comparison test of survival distributions for different levels of each group. F-censored and G-censored: when the study was completed on the 42nd day, there were alive rats in Group F (*n* = 3) and Group G (*n* = 8), but the survival time was not observed.

The survival analysis illustrated in Figure 1 indicated that the FRD-LC rats (Group D) have poorer survival rate when compared with the LC rats (Group B) (*P* < 0.01). The rats treated with the high dose of JPJD (Group G) survived longer compared with the other rats (*P* < 0.05). The FRD-LC rats (Group D) and the FRD-LC rats treated with the low dose of JPJD (Group E) survived for a shorter time compared with the other rats (*P* < 0.05). The LC rats (Group B) and the FRD-LC rats treated with the medium dose of JPJD (Group F) had an intermediate survival time. These results indicated that the FRD model was a promoting factor in the fatality rate of the FRD-LC model even when the FRD model was terminated, and the administration of 37.5 g·kg^−1^ JPJD could improve the survival rate of liver cancer (Figure 1).

42 days after transplantation with hepatic liver cancer, the weights of surviving rats in Groups B, D, E, and F continued to decrease, which was likely to be due to the growing hepatic tumor. However, the weights of rats in Group G decreased during the 28-day therapy but increased thereafter. Similarly, the numbers of surviving rats in the other groups were greater than the numbers of surviving rats in Groups B, D, E, and F during the experiment (*P* < 0.05, Table 1). The results of the survival analysis indicated that increased weight may improve the survival time.

### 3.2. Comparison of Spleen Weight, Liver Weight, Thymus Weight, Tumor Volume, and Tumor Weight in Each Group

Results of the tumor weight (Table 2) indicated that the tumor weight median of the FRD-LC group (Groups D) was the highest and the tumor weight median of the high dose of JPJD group (Group G) was the lowest. And the results of the tumor volume were consistent with the results of the tumor weight.

The organ weight of rats typically increases with increasing body weight. However, the rats analyzed here exhibited a lower organ weight index (organ weight/body weight), which did not accurately represent the organ weight changes. Thus, the actual organ weights were introduced to accurately reveal the effects of the interventions. The spleen and liver weights were significantly decreased in Groups B, D, and E (*P* < 0.05), and a smaller decrease was observed in Groups F and C. The spleen and liver weights of Group G were similar to Group A (*P* > 0.05). The thymus weights were higher in Group C and Group A and lower in Group B (*P* < 0.05) compared with all the other groups (*P* < 0.05), while the thymus weights were higher in Group G compared with Groups B, D, E, and F (*P* < 0.05) (Table 2).

These results indicated that the FRD model is a promoting factor in the growth of liver cancer, and the administration of 37.5 g·kg^−1^ JPJD could prevent the decline of organ weight in the FRD-LC model.

### 3.3. The Expression Levels of OATP1B2 and ABCC2

The regression coefficients for the standard curves of OATP1B2, ABCC2, and R-GAPDH were 0.996611, 0.995479, and 0.999462, while the slopes were 3.2010, 3.334250, and 3.398797, respectively. These data were confirmed and met the relative quantification requirements of a standard curve.

#### 3.3.1. Comparison of the Expression Levels of ABCC2 mRNA in the Liver and the Tumor Tissues among Different Groups (Figure 2). Expression Levels of OATP1B2 and ABCC2 mRNA in the Liver and Liver Cancer Tissues (Figures 2 and 3)

**Figure 2:**
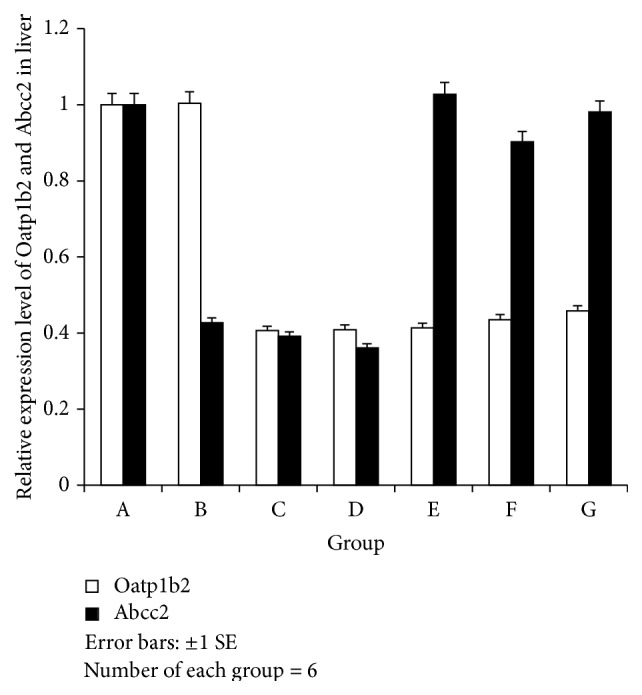
ABCC2 and OATP1B2 mRNA in liver. Versus A: ABCC2: B: *t* = −8.55, B: *P* = 0.013; C: *t* = 4.64, C: *P* = 0.01; D: *t* = 4.71; D: *P* = 0.042. OATP1B2: C: *t* = 5.90, C: *P* = 0.027; D: *t* = 4.46, D: *P* = 0.046; E: *t* = 5.56, E: *P* = 0.005; F: *t* = 2.92, F: *P* = 0.043; G: *t* = 4.41, G: *P* = 0.047.

Normal liver tissues highly expressed both ABCC2 and OATP1B2 mRNA. In the FRD, LC and FRD-LC rats, lower expression levels of ABCC2 mRNA were observed compared with the normal rats (*P* < 0.05). However, there was no difference between the expression level of ABCC2 mRNA of rats treated with JPJD and that of the normal rats (*P* > 0.05). The expression level of OATP1B2 mRNA of rats in Groups C, D, E, F, and G was much lower than that of the normal rats (*P* < 0.05) (Figure 2). These results indicated that OATP1B2 mRNA in the liver could be downregulated by FRD, FRD-LC, and JPJD, while ABCC2 mRNA could be downregulated by LC, FRD, and FRD-LC but upregulated by JPJD.

**Figure 3:**
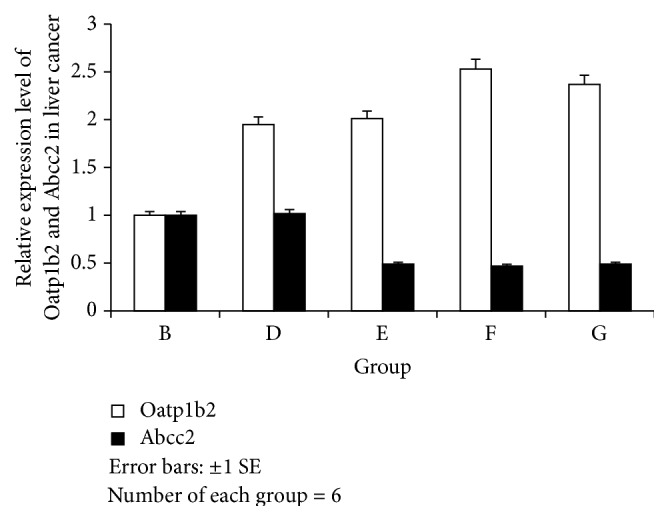
ABCC2 and OATP1B2 mRNA in liver cancer. Versus B: ABCC2: E: *t* = 26.1, E: *P* = 0.000; F: *t* = 30.9, F: *P* = 0.000; G: *t* = 27.9, G: *P* = 0.000; OATP1B2: D: *t* = −30.6, D: *P* = 0.000; E: *t* = −32.1, E: *P* = 0.000; F: *t* = −20.4, F: *P* = 0.000; G: *t* = −21.3, G: *P* = 0.000.

The liver cancer tissues in Group D expressed high levels of both ABCC2 and OATP1B2 mRNA. The rats treated with JPJD exhibited a higher expression level of OATP1B2 mRNA compared with the rats in Group B (*P* < 0.01). The rats treated with JPJD exhibited a lower expression level of ABCC2 mRNA compared with the rats in Group B (*P* < 0.01) (Figure 3). These results demonstrated that JPJD could upregulate the expression of OATP1B2 protein and downregulate ABCC2 protein in liver cancer tissues.

Compared with normal rats, the FRD and LC rats exhibited only slight effects on the ABCC2 mRNA levels in the liver separately, but the ABCC2 mRNA expression in the liver was significantly upregulated when the groups were combined (Figure 2A).

The expression level of ABCC2 mRNA in the liver of JPJD-treated FRD-LC rats (with a concentration-dependent trend) was lower than the FRD-LC rats that received no treatment and higher than the normal FRD and LC rats (Figure 2A). Additionally, a similar tendency of the expression of ABCC2 mRNA was observed in liver cancer tissue among these groups (Figure 2B).

#### 3.3.2. Comparison of the Expression Levels of OATP1B2 mRNA in the Liver and Tumor Tissues among Groups (Figure 2C and D)

Compared with the normal and LC rats, the FRD rats exhibited increased expression levels of OATP1B2 mRNA in the liver, while the FRD-LC rats that received no treatment exhibited the highest expression level of OATP1B2 mRNA among all the experimental groups (Figure 2C). However, the JPJD-treated FRD-LC rats (with a concentration-dependent trend) exhibited a lower expression level of OATP1B2 mRNA in the liver compared with the FRD rats and FRD-LC rats (Figure 2C). The expression level of OATP1B2 mRNA was the lowest in the FRD-LC rats compared with the other groups (Figure 2D). JPJD upregulated the expression level of OATP1B2 mRNA in the liver cancer tissues of FRD-LC rats with a concentration-dependent trend (Figure 2D).

#### 3.3.3. Expression Levels of OATP1B2 and ABCC2 Protein in the Liver and Liver Cancer Tissues (Figures 4 and 5)

**Figure 4:**
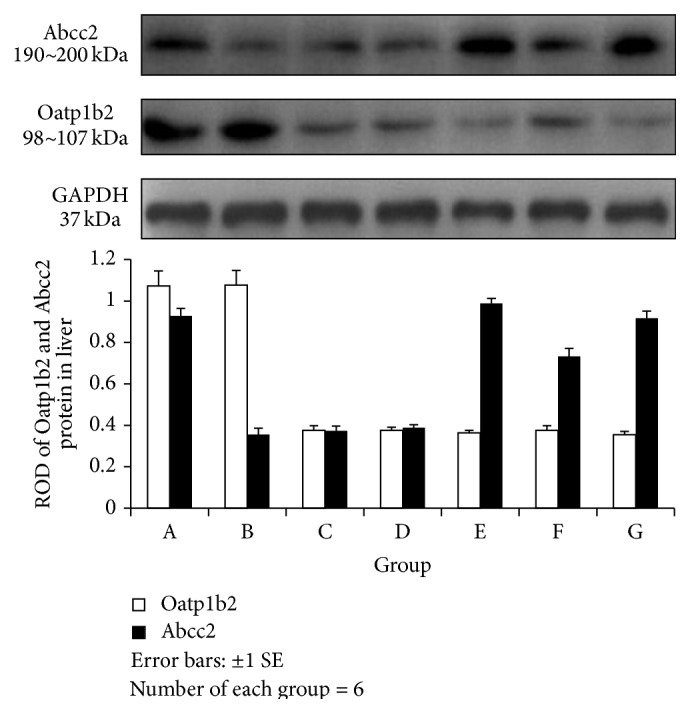
Relative optical density of ABCC2 and OATP1B2 in liver. ABCC2: one-way ANOVA tests revealed *F* = 4517.838, *P* = 0.000; A versus B, C, D, E, and F: *P* = 0.000; B, C, and D versus E, F, and G: *F* = 0.000. OATP1B2: one-way ANOVA *F* = 401.563, *P* = 0.000; A and B versus C, D, E, F, and G: *P* = 0.000.

Normal liver tissues highly expressed both ABCC2 and OATP1B2 proteins. However, the level of OATP1B2 was higher compared with ABCC2 in the LC model. In contrast, in the FRD and FRD-LC rats, lower expression levels of both ABCC2 and OATP1B2 proteins were observed compared with the normal rats. The expression level of ABCC2 protein was much higher than the expression level of OATP1B2 in the liver tissues of rats treated with JPJD (Figure 4). These results indicate that OATP1B2 proteins in the liver could be downregulated by FRD, FRD-LC, and JPJD, while ABCC2 proteins could be downregulated by LC, FRD, and FRD-LC but upregulated by JPJD.

**Figure 5:**
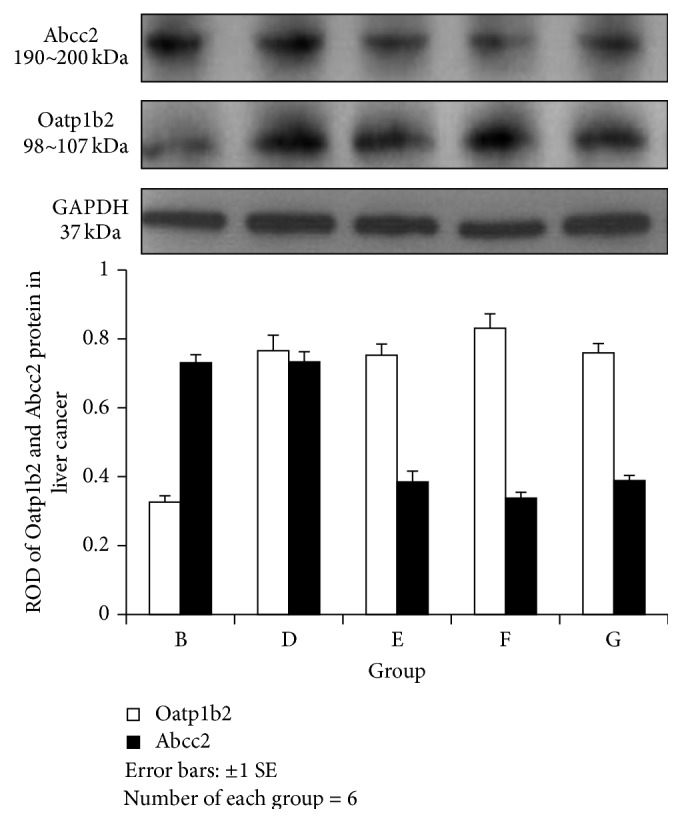
Relative optical density of ABCC2 and OATP1B2 in cancer. ABCC2: one-way ANOVA revealed *F* = 407.957, *P* = 0.000; B and D versus E, F, and G: *P* = 0.000; F versus E: *P* = 0.003 and G: *P* = 0.001. OATP1B2: one-way ANOVA revealed *F* = 203.479, *P* = 0.000; B versus D, E, F, and G: *P* = 0.000; F versus D: *P* = 0.004, E: *P* = 0.001; and G: *P* = 0.002.

The liver cancer tissues in Group D expressed high levels of both ABCC2 and OATP1B2 proteins. However, in the liver cancer tissues of LC rats, the expression level of the OATP1B2 protein was lower than the expression level of the ABCC2 proteins. The rats treated with JPJD exhibited a higher expression level of OATP1B2 protein compared with ABCC2 protein (Figure 5). These results demonstrated that JPJD could upregulate the expression of OATP1B2 protein and downregulate ABCC2 protein in liver cancer tissues. And the levels of both ABCC2 and OATP1B2 protein were almost consistent with the levels of mRNA.

### 3.4. Immunohistochemistry Results

**Figure 6:**
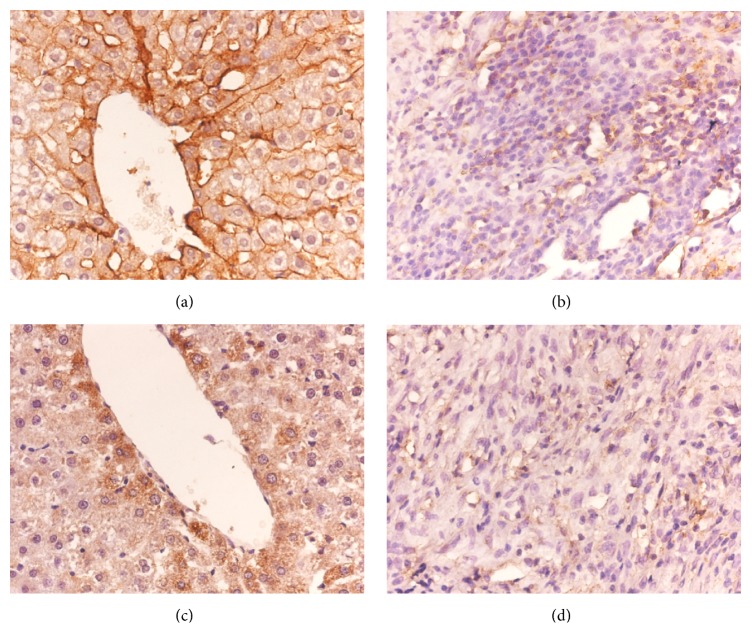
OATP1B2 and ABCC2 expression in liver and tumor tissue. (a) OATP1B2 expression in the liver cytoplasm and cell membrane (40x), (b) OATP1B2 expression in the tumor cytoplasm (40x), (c) ABCC2 expression in the liver cytoplasm (40x), and (d) ABCC2 expression in the tumor cytoplasm (40x).

OATP1B2 was highly expressed in the liver cell membrane of the liver tissue of the normal rats (Group A) (Figure 6(a)) and in the tumor tissue of the LC-induced rats (Group B). OATP1B2 was also expressed in the cytoplasm (Figure 6(b)). ABCC2 was expressed in the cytoplasm of the liver cells of the normal rats (Group A) with stronger expression in the cells closer to the central hepatic lobule compared with the cells farther away from the central vein (Figure 6(c)). ABCC2 was expressed in low level in liver tumor tissues (Figure 6(d)).

## 4. Discussion

Decreased food intake and diarrhea are the most common clinical symptoms in patients with liver cancer, and liver cancer with long-term decreased food intake or diarrhea usually shows rapid progression and poor prognosis. The impacts of reduced food intake and diarrhea on the development of liver cancer are still unclear. Studies in this field will be beneficial to providing an improved understanding of the clinical significance of liver cancer-associated gastrointestinal symptoms and providing basic evidence for the choice of therapeutic strategy in liver cancer.

The hepatic ABCC2 [30, 31] pump contributes to bile flow and is the main regulator of liver cancer chemoresistance [32–34]. Therefore, the expression of ABCC2 mRNA is upregulated during human hepatic carcinoma development [35, 36]. Previous studies have demonstrated that the liver-specific OATP1B2 in rodents is equivalent to the human liver-specific organic anion transporters OATP1B1 and OATP1B3 [37], expressed in human hepatocarcinoma [38] but highly expressed in the liver after liver transplantation [39, 40]. Thus, changes in the expression of ABC transporters and OATPs maybe lead to abnormal metabolism in the liver and liver cancer and alter the microenvironment of the liver.

In this study, a combined method of alternate-day fasting and laxative administration resulted in the symptoms such as slow weight gain, diarrhea, and lethargy in the rats. Although those symptoms disappeared after the model establishment factors were terminated in the FRD-LC model, the weight of the FRD-LC rats remained lower than that of the rats in the LC model group (Table 1). Moreover, the FRD-LC rats exhibited a higher tumor formation rate (Table 2) and a lower cumulative survival rate (Figure 1), which indicated that FRD could significantly reduce the anticancer ability of these rats. This adverse effect was maintained even after the FRD model establishment factors were terminated. So the study revealed that FRD might be detrimental to the survival time of LC-induced rats, and the stable body weight and higher organ weight might be beneficial to the survival of FRD-LC-induced rats. Compared with normal rats, the expression levels of ABCC2 in the liver tissue were decreased in the FRD group and the LC group. And the impacts of the lower level of the expression of ABCC2 from the FRD might be beneficial to the growth of the tumor in FRD-LC rats. This indicated that decreased food intake and diarrhea might be beneficial to the development of liver cancer. And the mechanism might be that FRD decreased the expression of ABCC2, so that the microenvironment was beneficial to the growth of liver cancer.

This study has confirmed that 37.50 g·kg^−1^ JPJD could improve the survival, weight changes, and organ weight changes of FRD-LC-induced rats. Although the details of the mechanism of JPJD is not clear, the metabolism-inducing and anticancer effects of the complex Chinese herb compound JPJD may contribute to its therapeutic effects based on the theory of Traditional Chinese Medicine. As mentioned previously, OATP1B2 is responsible for transporting extracellular substances into the cell, while ABCC2 is responsible for transporting intercellular substances out of the cell. Thus, the increased expression of ABCC2 and decreased expression of OATP1B2 in liver tissues and the decreased expression of ABCC2 and increased expression of OATP1B2 in cancer tissues caused by the administration of 37.50 g·kg^−1^ JPJD may be mechanisms by which JPJD treats liver cancer with food restriction and diarrhea. Additionally, ABCC2 is one of the major transmembrane transporter proteins contributing to drug resistance during chemotherapy. Therefore, future studies should explore whether JPJD can improve the internal environment during liver cancer and decrease drug resistance during chemotherapy.

## 5. Conclusions

Long-term food restriction and diarrhea may be an adverse factor for liver cancer. The Chinese Medicine JPJD is beneficial to the liver cancer rats with decreased food intake and diarrhea by upregulating the expression of the ABCC2 and downregulating the OATP1B2 in liver tissues while downregulating ABCC2 and upregulating OATP1B2 in cancer tissues. However, additional studies are needed to explore the therapeutic actions of JPJD and develop its specific clinical value.

## Figures and Tables

**Table 1:** Weight (median, g) changes and number (*N*) of rats left on different day.

Group	1st day (*N*)	7th day (*N*)	14th day (*N*)	21th day (*N*)	28th day (*N*)	35th day (*N*)	42th day (*N*)	Death day (*N*)	Weight loss
A	317 (15)	335 (15)	353 (15)	366 (15)	377 (15)	385 (15)	380 (15)	380 (15)	63
B	264 (15)	214 (15)	208 (15)	235 (11)	0 (5)	0 (0)	0 (0)	251 (15)	−13^a^
D	265 (15)	238 (15)	238 (15)	0 (4)	0 (1)	0 (1)	0 (0)	240 (15)	−25^a^
E	273 (15)	242 (15)	230 (15)	0 (5)	0 (1)	0 (0)	0 (0)	216 (15)	−57^a^
F	269 (15)	261 (15)	233 (15)	218 (13)	0 (4)	0 (3)	0 (3)	223 (15)	−46^a^
G	259 (15)	251 (15)	229 (15)	233 (13)	253 (10)	265 (8)	282 (8)	301 (15)	42

The weight loss = median weight when rats were sacrificed − median weight weighted when liver cancer model was created. Independent sample rank sum tests for weight loss revealed *P* < 0.01; ^a^ *P* < 0.01  versus G and A.

**Table 2:** Comparison for weight of organs (mean ± SD, g), volume of tumor (median, cm^3^), and weight of tumor (median, g).

Group	*N*	Spleen	Liver	Thymus	Tumor volume	Tumor weight
A	15	0.58 ± 0.04^a,b,d^	11.26 ± 1.76	0.42 ± 0.07^c^	—	—
B	15	0.33 ± 0.08	7.49 ± 1.57^a,c^	0.19 ± 0.09^a^	1.41	1.06
C	15	0.48 ± 0.03^a,b^	8.43 ± 0.58^b^	0.45 ± 0.08^b^	—	—
D	15	0.33 ± 0.08	7.93 ± 1.24^a,c^	0.21 ± 0.09^a^	2.41	1.93
E	15	0.37 ± 0.07	8.14 ± 1.16^b^	0.22 ± 0.07^e^	2.01	1.63
F	15	0.42 ± 0.09	8.21 ± 1.28^b^	0.27 ± 0.10	1.96	1.57
G	15	0.51 ± 0.13^a,c^	9.59 ± 1.36	0.38 ± 0.09	0.75	0.56

Median tumor volume = the longest diameter × the shortest diameter^2^/2.

A one-way ANOVA test revealed values of *F* = 15.18 and *P* = 0.000 for spleen weight, *F* = 10.47 and *P* = 0.000 for liver weight, and *F* = 19.34 and *P* = 0.000 for thymus weight. The levels of significance were as follows. For spleen weight: ^a^ *P* < 0.01  versus B, D; ^b^ *P* < 0.01, ^c^ *P* < 0.05  versus  E. For liver weight, ^a^ *P* < 0.01, ^b^ *P* < 0.05  versus A; ^c^ *P* < 0.05  versus G. For thymus weight, ^a^ *P* < 0.01  versus A, C, and G; ^b^ *P* < 0.01, ^c^ *P* < 0.05  versus  F. Independent sample rank sum tests for tumor volume and tumor weight among these groups revealed *P* > 0.05.
